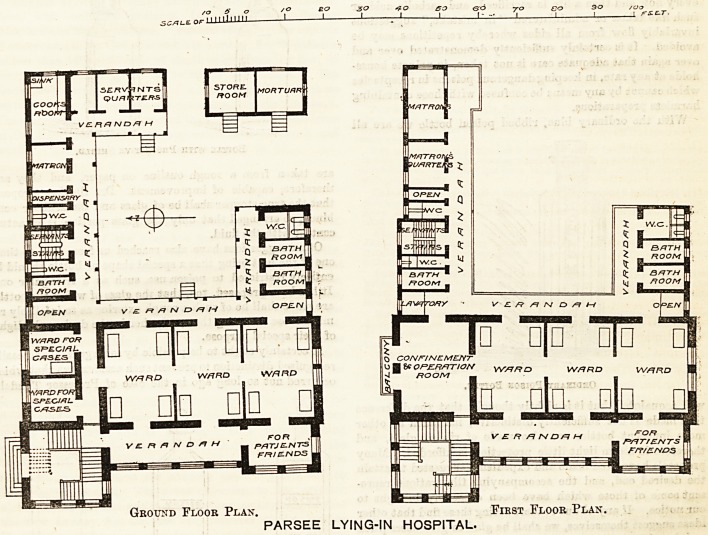# The Parsee Lying-in Hospital, Bombay

**Published:** 1895-08-24

**Authors:** 


### The Institutional Workshop.

### HOSPITAL CONSTRUCTION.

### THE PARSEE LYING-IN HOSPITAL, BOMBAY.

This institution, which was opened early in the present year by the then Governor of Bombay, Lord Harris, replaces a smaller building not specially adapted for the purpose, but in which for many years much useful work was done. Dr. Nariman, to whose energy the establishment of the hospital on a permanent basis is mainly due, finding an abnormal prevalence of puerperal fever amongst the women of the Parsee community, set himself to investigate the etiology of the disease. He found that, while the existence of certain insanitary social customs undoubtedly lent their aid to propagation of disease, an important factor in its production was unquestionably exposure to contact with sewer air. And, in the face of much opposition, he proved that puerperal fever was preventable, and that, in fact, in 1,100 cases of all kinds treated in the old Lying-in Hospital not a single case of fever occurred, while the mortality was barely one-half per cent.

The conditions under which Parsee women are, as a rule, confined, appear to be the very reverse of sanitary. Dark, fetid, ill-ventilated rooms on the ground floor in close proximity to the latrines, with a crowd of female friends and sympathisers present, and with customs regarding linen, &c., the reverse of aseptic; such are the surroundings of a Parsee mother. It can scarcely be matter for surprise that the death-rate of the community from puerperal fever should reach an abnormal figure.

The new hospital is planned on a system which involves the freest possible movement of air around all parts of the building. All the rooms open on to verandahs, which not only serve to keep the various wards, &c., cool, but secure the effectual severance between the air of the wards and that of the sanitary offices.

The ground floor contains four wards of four beds each, all communicating, with two single bed wards for special cases.

Ground Floor Plan. First Floor Plan.

PARSEE LYING-IN HOSPITAL.

The wings are separated from the ward building by open lobbies, in continuation of the verandah. In the larger wing, besides a bath-room and w.c., are the dispensary, matron's room, kitchen, offices, and servants' rooms. The smaller wing contains two bath-rooms and w.c.'s. The first floor is a repeat of the ground floor, except that a confinement and operating-room occupies the space over the two small wards and passage on the floor below. The only questionable arrangement is the door which leads from the confinement-room to the ward. It would surely have been safer to have had no entrance into the confinement-room except from the staircase on one side and the open verandah on the other.

A second floor is formed over a part of the building, and contains a ward for four beds, with bath-room and w.c., and part of the roof over the remaining portion is formed into a terrace.

The buildings were designed by and erected under the superintendence of Khan Bahadur M. C. Murzban, C.I.E., the executive officer of the Bombay Municipality, and afford accommodation for forty patients, which, it is stated, may be increased to fifty by utilising the verandahs.

## Figures and Tables

**Figure f1:**